# Origin and spread of human mitochondrial DNA haplogroup U7

**DOI:** 10.1038/srep46044

**Published:** 2017-04-07

**Authors:** Hovhannes Sahakyan, Baharak Hooshiar Kashani, Rakesh Tamang, Alena Kushniarevich, Amirtharaj Francis, Marta D Costa, Ajai Kumar Pathak, Zaruhi Khachatryan, Indu Sharma, Mannis van Oven, Jüri Parik, Hrant Hovhannisyan, Ene Metspalu, Erwan Pennarun, Monika Karmin, Erika Tamm, Kristiina Tambets, Ardeshir Bahmanimehr, Tuuli Reisberg, Maere Reidla, Alessandro Achilli, Anna Olivieri, Francesca Gandini, Ugo A. Perego, Nadia Al-Zahery, Massoud Houshmand, Mohammad Hossein Sanati, Pedro Soares, Ekta Rai, Jelena Šarac, Tena Šarić, Varun Sharma, Luisa Pereira, Veronica Fernandes, Viktor Černý, Shirin Farjadian, Deepankar Pratap Singh, Hülya Azakli, Duran Üstek, Natalia Ekomasova (Trofimova), Ildus Kutuev, Sergei Litvinov, Marina Bermisheva, Elza K. Khusnutdinova, Niraj Rai, Manvendra Singh, Vijay Kumar Singh, Alla G. Reddy, Helle-Viivi Tolk, Svjetlana Cvjetan, Lovorka Barac Lauc, Pavao Rudan, Emmanuel N. Michalodimitrakis, Nicholas P. Anagnou, Kalliopi I. Pappa, Maria V. Golubenko, Vladimir Orekhov, Svetlana A Borinskaya, Katrin Kaldma, Monica A. Schauer, Maya Simionescu, Vladislava Gusar, Elena Grechanina, Periyasamy Govindaraj, Mikhail Voevoda, Larissa Damba, Swarkar Sharma, Lalji Singh, Ornella Semino, Doron M. Behar, Levon Yepiskoposyan, Martin B. Richards, Mait Metspalu, Toomas Kivisild, Kumarasamy Thangaraj, Phillip Endicott, Gyaneshwer Chaubey, Antonio Torroni, Richard Villems

**Affiliations:** 1 Evolutionary Biology Group, Estonian Biocentre, Tartu 51010, Estonia; 2 Laboratory of Ethnogenomics, Institute of Molecular Biology of National Academy of Sciences, Yerevan 0014, Armenia; 3 Dipartimento di Biologia e Biotecnologie “L. Spallanzani”, Università di Pavia, Pavia 27100, Italy; 4 Department of Zoology, University of Calcutta, Kolkata 700 019, India; 5 Institute of Genetics and Cytology, National Academy of Sciences, Minsk 220072, Belarus; 6 CSIR-Centre for Cellular and Molecular Biology, Hyderabad 500 007, India; 7 Faculty of Biological Sciences, University of Leeds, Leeds LS2 9JT, UK; 8 Instituto de Patologia e Imunologia Molecular da Universidade do Porto (IPATIMUP), Porto 4200-135, Portugal; 9 Department of Evolutionary Biology, Institute of Molecular and Cell Biology, University of Tartu, Tartu 51010, Estonia; 10 Human Genetics Research Group, Department of Biotechnology, Shri Mata Vaishno Devi University, Katra 182320, India; 11 Utrecht 3523 GN, The Netherlands; 12 Russian-Armenian University, Yerevan 0051, Armenia; 13 Department of Medical Genetics, National Institute of Genetic Engineering and Biotechnology, Tehran 14965/161, Iran; 14 Departamento de Biologia, CBMA (Centro de Biologia Molecular e Ambiental), Universidade do Minho, Braga 4710-057, Portugal; 15 Institute for Anthropological Research, Zagreb 10000, Croatia; 16 Instituto de Investigação e Inovação em Saúde, Universidade do Porto (i3S), Porto 4200-135, Portugal; 17 Department of Anthropology and Human Genetics, Faculty of Science, Charles University, Prague 128-43, Czech Republic; 18 Department of Immunology, Allergy Research Center, Shiraz University of Medical Sciences, Shiraz 71348-45794, Iran; 19 Genetic Department, Institute of Experimental Medicine, Istanbul University, Istanbul 33326, Turkey; 20 Department of Medical Genetics and REMER, Faculty of Medicine, Medipol University, Istanbul, 34810 Turkey; 21 Institute of Biochemistry and Genetics, Ufa Scientific Center of the Russian Academy of Sciences, Ufa 450054, Russia; 22 Department of Genetics and Fundamental Medicine of Bashkir State University, Ufa 450076, Russia; 23 Department of Molecular Biology, Ruđer Bošković Institute, Zagreb 10000, Croatia; 24 Mediterranean Institute for Life Sciences, Split 21000, Croatia; 25 Croatian Science Foundation, Zagreb 10000, Croatia; 26 Anthropological Centre of the Croatian Academy of Sciences and Arts, 10000 Zagreb, Croatia; 27 Department of Forensic Sciences and Toxicology, University of Crete, School of Medicine, Heraklion 71110, Greece; 28 Laboratory of Biology, University of Athens, School of Medicine, Athens 115 27, Greece; 29 Foundation for Biomedical Research of the Academy of Athens (IIBEAA), Athens 115 27, Greece; 30 First Department of Obstetrics and Gynecology, University of Athens, School of Medicine, Athens 115 27, Greece; 31 Research Institute of Medical Genetics, Tomsk National Research Medical Center of the Russian Academy of Sciences, Tomsk 634050, Russia; 32 Vavilov Institute of General Genetics, Russian Academy of Sciences, Moscow 119333, Russia; 33 Institute of Cellular Biology and Pathology “Nicolae Simionescu”, Bucharest PO Box 35-14, Romania; 34 Kharkiv Specialized Medical Genetic Centre (KSMGC), Kharkiv 61022, Ukraine; 35 Institute of Internal and Preventive Medicine, SB RAS, Novosibirsk 630089, Russia; 36 Institute of Cytology and Genetics SB RAS, Novosibirsk 630090, Russia; 37 Novosibirsk State University, Novosibirsk 630090, Russia; 38 Clalit National Cancer Control and Personalized Medicine Program, Carmel Medical Center, Haifa 3436212, Israel; 39 Department of Biological Sciences, School of Applied Sciences, University of Huddersfield, Huddersfield HD1 3DH, United Kingdom; 40 Department of Archaeology and Anthropology, University of Cambridge, Cambridge CB2 1QH, United Kingdom; 41 Musée de l’Homme, Paris 75116, France; 42 Estonian Academy of Sciences, Tallinn 10130, Estonia

## Abstract

Human mitochondrial DNA haplogroup U is among the initial maternal founders in Southwest Asia and Europe and one that best indicates matrilineal genetic continuity between late Pleistocene hunter-gatherer groups and present-day populations of Europe. While most haplogroup U subclades are older than 30 thousand years, the comparatively recent coalescence time of the extant variation of haplogroup U7 (~16–19 thousand years ago) suggests that its current distribution is the consequence of more recent dispersal events, despite its wide geographical range across Europe, the Near East and South Asia. Here we report 267 new U7 mitogenomes that – analysed alongside 100 published ones – enable us to discern at least two distinct temporal phases of dispersal, both of which most likely emanated from the Near East. The earlier one began prior to the Holocene (~11.5 thousand years ago) towards South Asia, while the later dispersal took place more recently towards Mediterranean Europe during the Neolithic (~8 thousand years ago). These findings imply that the carriers of haplogroup U7 spread to South Asia and Europe before the suggested Bronze Age expansion of Indo-European languages from the Pontic-Caspian Steppe region.

Ancient DNA (aDNA) studies in the last decade or so have substantially broadened our knowledge of prehistoric human demography, revealing major population turnovers in Europe during the Holocene^1^,^2^,^3^ and the late Pleistocene^4^,^5^. Out of two pan-Eurasian mitochondrial DNA (mtDNA) founder lineages (M and N)^6^, the majority of contemporary Europeans and Southwest Asians cluster into macro-haplogroup N (including its major subclade R)^7^,^8^. Haplogroup (hg) U – a sub-branch of hg R – shows a wide distribution in both regions. Its Upper Palaeolithic presence in Europe was initially recognized on the basis of modern-day population data^7^,^9^,^10^, and confirmed by aDNA studies, which revealed that various subclades of hg U encompassed the vast majority of European mitogenomes during the Palaeolithic and Mesolithic, and that most of the other (non-U) mtDNA lineages appeared only later in the Holocene^1^,^2^,^4^,^5^. On the other hand, phylogeographic surveys of modern mitogenomes of hgs I, W, J and T have identified signals of Late Glacial/postglacial expansions from the Near East to Europe, thus implying that the presence in Europe of these Near Eastern haplogroups predated the Neolithic^11^,^12^,^13^, but these haplogroups have not been detected so far in pre-Neolithic human remains.

Hg U is subdivided into U1, U5, U6, and a fourth subclade, which further divides into U2, U3, U4′9, U7, and U8 (including hg K). Many of these U subclades display region-specific frequency patterns in present-day populations: hgs U1 and U3 are largely restricted to the Near East^14^,^15^,^16^, U4 and U5 to Europe^7^,^9^,^17^,^18^, U6 to the circum-Mediterranean region, with a frequency peak in North Africa^19^,^20^,^21^, while U8 is more prevalent in the Near East and Europe^7^,^22^,^23^,^24^,^25^ and U9 is rare with only sporadic occurrences in Arabia, Ethiopia and India^26^,^27^. Hg U2 harbours frequency and diversity peaks in South Asia, whereas its subclades U2d and U2e are confined to the Near East and Europe^25^,^28^,^29^,^30^.

Compared to other subclades of hg U, both the phylogenetic structure and the ancestral origin of hg U7 are rather obscure. This haplogroup is characterized by generally low population frequencies and limited sequence diversity, despite a geographic distribution ranging from Europe to India^14^,^16^,^25^,^27^,^30^,^31^,^32^,^33^. Recently, it has been detected in skeletal remains from Southwest Iran dated ~six thousand years ago (kya)^34^ as well as in remains from the Tarim Basin in Northwest China (3.5–4.0 kya)^35^.

It has been previously shown that low-frequency mitochondrial haplogroups with relict distributions, similar to hg U7, can be disproportionately informative about ancient human dispersal events^36^,^37^,^38^. Although, mtDNA itself, as a single locus, often does not reflect the whole complexity of past demographic processes^39^,^40^,^41^,^42^, detailed phylogenies and phylogeographic surveys based on a large number of thoroughly collected and sequenced mitogenomes might provide unique insights on gender-specific gene flows, not always obvious from genome-wide studies, and on contrasting patterns of patri- and matrilineal heritage, as well as reliable time estimates. To evaluate whether high-resolution phylogeographic data from hg U7 could provide new clues on the prehistory and ancestral origins of the modern-day populations that currently harbour this haplogroup, we first assembled a large number (1141) of control-region sequences (Supplementary Table S1) and then sequenced 267 U7 mitogenomes from its entire distribution range.

## Results and Discussion

**Figure 1:**
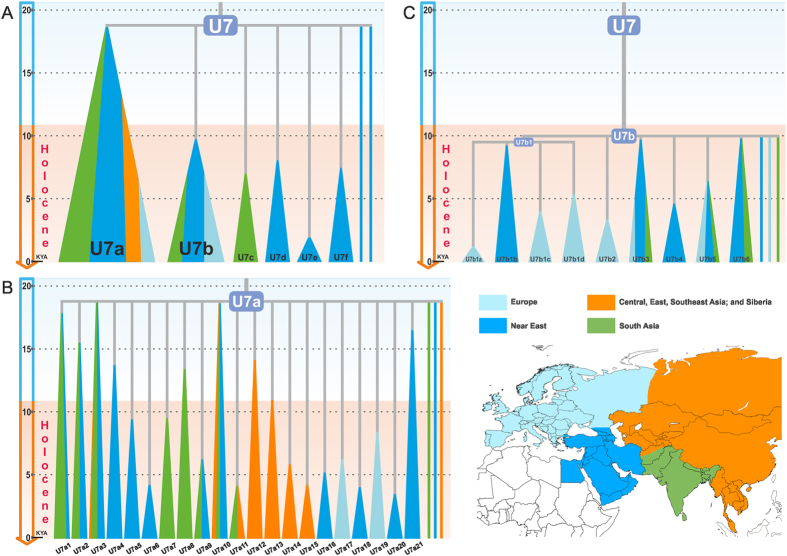
Schematic Representations of the U7, U7a and U7b Phylogenies. Subclades are represented by triangles, while single lineages are represented by lines. Subclades and single linage lines are colored according to their geographic origin, as shown in the map (lower right corner). (**A**) U7 tree. (**B**) U7a tree. (**C**) U7b tree. KYA – thousand years ago. Map was generated with Surfer program (version 8, Golden Software, Inc., Golden, CO, USA, https://www.goldensoftware.com/).

The maximum-parsimony reconstruction of 367 sequences of hg U7 yielded a tree with a basal hard polytomy that cannot be resolved (to a dichotomous one) at the level of whole-mtDNA sequence data: we identified eight independent branches that coalesce at the root of U7 (Fig. 1A and Supplementary Figure S1). Consistent with previous studies, we found that three major branches, U7a–c, capture most (96%) of the U7 mitogenomes. Besides these three previously known branches, we identified three additional clades, hereby designated as U7d, U7e, and U7f (Table 1). These were exclusively seen in Iran and the Caucasus. Finally, two mitogenomes – also from Iran and the Caucasus – did not cluster with any of hgs U7a–f and remained as unlabelled single lineages.

**Figure 2:**
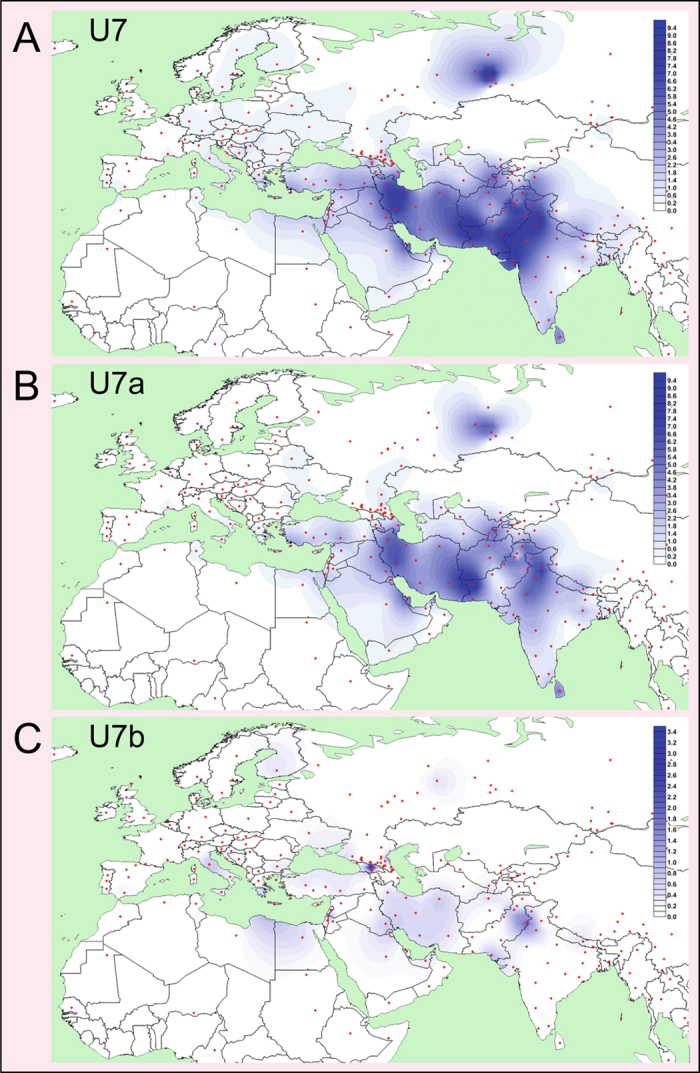
Spatial Frequency Distribution Maps of Haplogroups U7, U7a and U7b. Dots indicate the geographical locations of the surveyed populations. Population frequencies (%) correspond to those listed in Supplementary Table S3. Note the different frequency scales used in different maps. Maps were generated with Surfer program (version 8, Golden Software, Inc., Golden, CO, USA, https://www.goldensoftware.com/).

In agreement with previous observations^16^, U7c appears to be restricted to South Asia (Fig. 1A and Supplementary Figure S1). In contrast, U7a is the dominant branch of U7 throughout the Near East and South Asia with subclades specific to Central Asia (U7a12–15), Mediterranean and Southeast Europe (U7a17 and U7a19; Figs 1B and 2B, Supplementary Figure S1). U7b exhibits a higher frequency than U7a in Europe with elevated levels of diversity in the Mediterranean and southeastern regions (Figs 1C and 2C and Supplementary Figure S1). It is distributed also in the Near East, South and Central Asia.

We estimated a coalescence time for hg U7 at ~15.6–18.6 kya (Table 1), in agreement with previous maximum-likelihood estimates^31^. This confirms that U7 is the youngest major clade within the macro-hg U and the only one with the most recent common ancestor after the Last Glacial Maximum (LGM), presumably resulting from a severe glacial bottleneck. All other hg U subclades (U1, U2, U3, U4′9, U5, U6, and U8) display considerably older ages (~30–43 kya)^31^. This loss of genetic diversity of the ancestral U lineage, which eventually led to the formation of hg U7 is consistent with the survival of a small number of founders during the LGM; a pattern similar to that observed for mtDNA hgs N1a3 (previously N1c), N3, W, R2, HV, and within hgs M1 and U6^11^,^16^,^20^,^21^,^43^,^44^,^45^.

**Figure 3:**
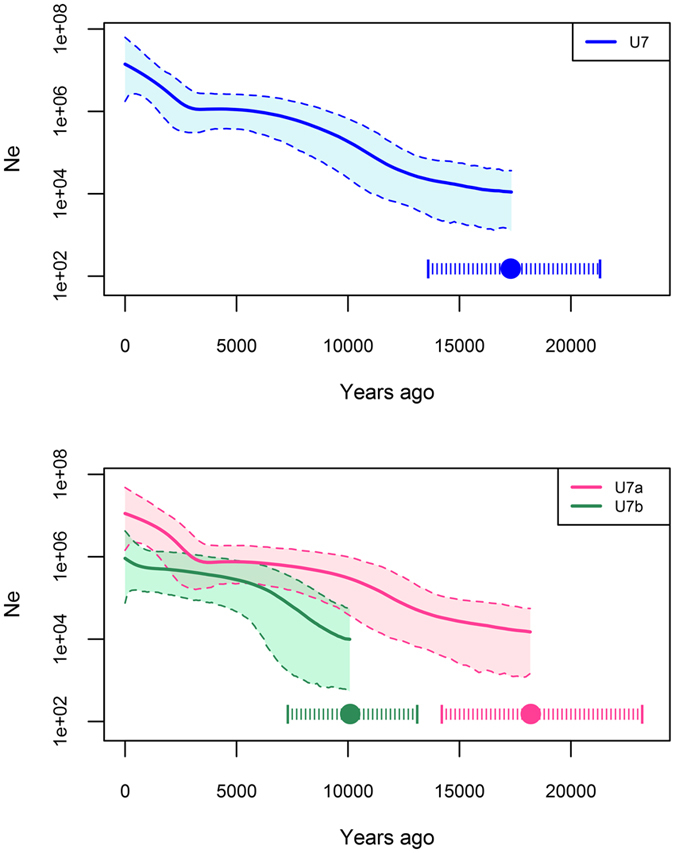
Bayesian Skyline Plots of Haplogroups U7, U7a and U7b. The solid line is the median estimate, while dashed lines show the 95% highest posterior density (HPD) limits. Means (filled circles) and HPD intervals (pipes) for coalescence times are provided in the figure with corresponding colors. *N*_*e*_: effective population size.

The hg U7 Bayesian skyline analysis (Fig. 3) shows a clear signal for an overall demographic expansion after the LGM. U7a drives the early stages of this demographic expansion, whereas the signal for U7b (the predominantly European subclade of hg U7) occurs much later, ~8–5 kya (Table 1 and Fig. 3). The subclades of U7a that are common in the Near East and South Asia (U7a1, U7a2, U7a3, and U7a10) are characterized by coalescence dates and a growth phase prior to the Holocene (Supplementary Figure S1 and Supplementary Table S4). Among those, U7a3 is both the oldest (~19 kya) and most frequent throughout these two areas, whilst U7a1, U7a2 and U7a10 are older than 12 kya. Clades U7a2, U7a3 and U7a10 have individual components, specific to the Near East and South Asia, suggesting that U7a was already differentiated in both regions by the end of the Pleistocene.

Central Asia has four regionally specific clades (U7a12–15), whilst U7a11 is shared with South Asia (Fig. 1B and Supplementary Figure S1). U7 is distributed unevenly in Central Asia; it is most frequent in its southern areas adjacent to the Near East and South Asia (Fig. 2), with Afghanistan forming a “buffer zone”. Despite this geographical proximity, there are very few instances of shared lineages among the three regions, which, combined with the early coalescence date for U7a12 (~12 kya) (Supplementary Table S4), is consistent with a regional differentiation prior to the Holocene.

In contrast to U7a, U7b shows signs of a significantly (t-test p < 0.001) later expansion and is characterized by low frequencies in the Near East, South Asia, and Central Asia, while it has a higher frequency in Europe (Table 1, Supplementary Tables S3 and S4). This differentiation is also reflected in the number of U7b subclades that are restricted to Europe – four out of nine subclades identified in the current phylogeny (Fig. 1C and Supplementary Figure S1). In addition, many single lineages (eight out of eighteen) in U7b are from Europe. The major sub-branches of U7b are characterized by star-like radiations and growth ~8–5 kya (Fig. 3). Subclades U7b1c and U7b1d, which are exclusive to Europe, expanded ~5 kya (Supplementary Figure S1). Hence, we consider this time as a minimum age for the presence of U7b in Europe. Taking into account the U7b coalescence age in Europe (Table 1), we think that U7b may have appeared there between 5 and 10 kya. This timeframe overlaps significantly with the time of the Neolithic demographic transition in Europe.

Expansions within this timeframe are also observed for the European-specific clades U7a17 and U7a19, whose distributions are centred on Mediterranean and Southeast Europe, along one of the preferred routes for the initial dispersal of farming^46^,^47^,^48^,^49^. Elevated frequencies in the Mediterranean area are also witnessed for many subsets of U7b (Fig. 2C), and the age of U7b in Europe (5–10 kya) is incompatible with its presence there prior to the Holocene. To date, aDNA studies have not found any example of U7 in either Neolithic or pre-Neolithic contexts^1^,^4^,^5^,^50^,^51^,^52^,^53^,^54^,^55^,^56^,^57^,^58^. In contrast, other clades of hg U were common during the postglacial re-expansion period, including U8a, which is extremely rare today^24^. However, the number of analysed ancient samples from the Mediterranean area, where U7b has elevated frequencies today, is still rather small.

The reduction in frequency of U7 in Europe from south to north is mirrored by the main components of hg K (a sister clade of U8b1). These have also been argued to have arrived into Europe during the early Neolithic from the Near East^2^,^59^,^60^,^61^, and display a clear northward frequency cline^23^,^62^. According to aDNA evidence, Neolithic populations in Europe display a distinct mtDNA lineage make-up, argued to be derived from Near Eastern sources^1^,^5^,^50^,^51^,^52^,^55^,^56^,^57^,^58^,^63^. This early colonisation was probably followed by a complex process of assimilation of autochthonous hunter-gatherer diversity, seen most clearly in the autosomes. Notably, the distribution of nuclear genetic variants from Neolithic migrants among modern-day European populations^3^,^55^,^64^,^65^,^66^,^67^,^68^,^69^,^70^,^71^ resembles the phylogeography of hg U7 in Europe today.

Another major episode of gene flow affecting the European gene pool appears to have occurred during the Late Neolithic and Early Bronze Age, from a source in the Pontic-Caspian Steppe region north of the Caucasus^3^,^54^,^66^,^72^. It has been suggested that this migration resulted in a further substantial shift in the genetic profile of Europeans and was a major vehicle for the movement of Indo-European languages to Europe^3^,^72^, and likely also to South Asia^54^. Interestingly, the autosomal genetic component in Europeans considered to derive from the Steppe is almost fixed in two pre-Neolithic ancient genomes from the South Caucasus. This component is distributed eastwards towards South Asia as well^54^, where it mimics the distribution of U7 (Pearson’s r = 0.65, p = 0.01). Our time estimates for the expansion and differentiation of hg U7 in the Near East, Central Asia, South Asia, and Europe, however, predate these putative late Neolithic-early Bronze Age migrations and thereby rule them out as a major vehicle for the spread of U7 to Europe and South Asia. In this respect, it is also noteworthy that Yamnaya herders of the Steppe so far analysed (n = 43) show no traces of U7^3^,^55^,^72^,^73^ – and U7 is rarely found in this region today (Fig. 2).

The expansion time of hg U7 in the Near East, Central Asia and South Asia is more consistent with autosomal multi-locus estimates for the genetic separation of these regions during the Terminal Pleistocene^74^, suggesting a common demographic process, whose origin was unclear previously. Here, we show that the frequency and distribution of U7b lineages indicate an origin of this clade in the Near East, whilst for U7a these statistics cannot differentiate between South Asia and the Near East (including the Caucasus) as a possible homeland. Within the Near East hg U7 is most frequent and diverse in Iran, whilst in South Asia its frequency and diversity peaks are in the Indus Valley region (Fig. 2 and Supplementary Figure S1). The demographic histories of the Near East and South Asia show marked differences during the LGM with the latter being less affected^75^. This is consistent with the long-term high effective population size and deep structure of autochthonous mtDNA haplogroups in South Asia^76^,^77^,^78^, in striking contrast to the severe population reduction affecting hg U7 during the last glacial period. Conversely, hg U2 in South Asia dates to more than 35 kya^31^, indicating its presence prior to the LGM. Moreover, other haploid (Y chromosomal hg J2)^79^ and diploid^80^,^81^ genetic markers provide support for a post-glacial dispersal from the Near East to South Asia before the Bronze Age. Interestingly, recent aDNA studies have revealed a significant shared ancestry between Neolithic populations from the Zagros Mountains in Iran and contemporary populations from South Asia, suggesting eastward migration of people from that region to South Asia already at least in the Neolithic timeframe^34^,^82^.

In conclusion, the Near East is the most likely ancestral homeland of U7. Our analyses reveal two temporally and geographically distinct signals of U7 expansion that disseminated from this region. The first signal dates shortly after the LGM and this dispersal is responsible for the spread of U7 towards South and Central Asia prior to the Holocene, while the more recent expansion explains its spread in Mediterranean Europe most probably during the early Holocene. These dispersals of hg U7 towards South Asia and Europe preclude any major association of U7 with the putative Bronze Age expansion of the Indo-European language family to these regions.

## Materials and Methods

The sampling encompassed the Near East, South Asia, Europe and Central Asia (Supplementary Table S2). For the purposes of this study, both Near East and Southwest Asia refer to the territory that includes the Levant, Anatolia, Caucasus, Iraq, Iran, Arabia, and Lower Egypt. U7 samples were selected from mtDNA databases of the research groups involved in this study. Blood specimens were collected from healthy unrelated adult individuals whose matrilineal ancestors for at least two generations belonged to the populations reported here. Informed consent was obtained from all participants in the study. All experimental procedures were carried out in accordance with the approved guidelines by the Research Ethics Committee of the University of Tartu and the Ethic Committee for Clinical Experimentation of the University of Pavia. All experimental protocols were approved by the Research Ethics Committee of the University of Tartu (252/M-17) and the Ethic Committee for Clinical Experimentation of the University of Pavia (Board minutes of the October 5, 2010). The mitogenome sequencing was carried out following published protocols^83^,^84^. Mutations were scored relative to the Reconstructed Sapiens Reference Sequence (RSRS)^31^ and the Revised Cambridge Reference Sequence (rCRS)^85^,^86^. For these tasks as well as for sequence alignments the following software packages were used – ChromasPro (Technelysium Pty Ltd, South Brisbane QLD 4101, Australia), mtDNACommunity^31^, and BioEdit^87^. A maximum-parsimony tree was constructed using a total of 367 hg U7 mitogenomes (267 new from this study and 100 from the literature), guided by published principles^88^ (Supplementary Figure S1 and Supplementary Table S2). In addition, the control region and/or phylogenetically informative markers from the coding region were sequenced for 229 samples (Supplementary Table S1). This, together with the re-constructed high-resolution phylogeny, has allowed us to assign into major branches almost 85% of all U7 samples available from the literature and our collection (Supplementary Table S1). Spatial frequency maps were generated with Surfer program (version 8, Golden Software, Inc., Golden, CO, USA), following the Kriging algorithm (input data is represented in Supplementary Table S3). Coalescence times were calculated using the rho (ρ) statistic^89^ and standard deviations^90^. Genetic distances were calculated with both the complete and synonymous clock models and converted into years using the published calculator^91^. We used this mutation rate and the calculator because of the evidence of nonlinearity in human mtDNA mutation rate, and the necessity for correction of time estimates for purifying selection^91^,^92^. Coalescence time estimates were also computed with the Bayesian MCMC approach implemented in the BEAST v1.7.5 suite of software^93^, using five partitions of the mtDNA genome: control region, tRNA plus rRNA regions, first, second, and third positions of codons in the protein coding regions. Good convergence was achieved by applying the HKY^94^ and strict clock models^95^; hence all Bayesian analyses in this study were carried out using these models. A Bayesian skyline model was used as the tree model^96^. An average value (1.6865E-8 substitutions/site/year) of two published whole-mtDNA mutation rates (1.665E-8 and 1.708E-8)^91^ was used as a prior in the analyses. Some of the BEAST runs were carried out in the CIPRES public resource^97^. Bayesian skyline analyses were carried out with Tracer software v1.6. As the BEAST v1.7.5 software assumes a linear mutation rate, we corrected time estimates obtained from BEAST v1.7.5 analyses as well as for the Bayesian skyline plots by the published formula^91^. Updated skyline plots were generated using the R software (the R project) with the basic packages.

## Additional Information

**Accession codes:** The previously unreported 267 mitogenome sequences have been deposited in GenBank (http://www.ncbi.nlm.nih.gov/genbank/) under accession numbers KY824818-KY825084.

**How to cite this article**: Sahakyan, H. *et al*. Origin and spread of human mitochondrial DNA haplogroup U7. *Sci. Rep.* **7**, 46044; doi: 10.1038/srep46044 (2017).

**Publisher's note:** Springer Nature remains neutral with regard to jurisdictional claims in published maps and institutional affiliations.

## Supplementary Material

Supplementary Figure and Table Legends

Supplementary Figure S1

Supplementary Dataset 1

Supplementary Dataset 2

Supplementary Dataset 3

Supplementary Dataset 4

## Figures and Tables

**Table 1:** Age Estimates, Defining Mutations, and Distribution Ranges of Haplogroup U7 and Its Subclades.

Clade	Defining Mutations	Geographic Region	Age Estimates (kya)		
			Rho Complete	Rho Synonymous	BEAST, Corrected
U7	T152C, T980C, C3741T, C5360T, C8137T, C8684T, C10142T, T13500C, G14569A, A16309G, A16318t	All together	18.6 (13.6–23.7)	15.6 (11.0–20.3)	17.3 (13.6–21.3)
		Near East	—	—	15.4 (11.9–19.3)
		South Asia	—	—	15.6 (12.2–19.7)
		Europe	—	—	13.2 (9.3–13.2)
		Central Asia	—	—	16.3 (11.2–22.8)
U7a	C151T	All together	18.7 (14.9–22.7)	19.2 (11.4–26.9)	18.2 (14.2–23.2)
		Near East	—	—	15.3 (11.5–19.3)
		South Asia	—	—	17.0 (12.6–21.9)
		Europe	—	—	17.1 (11.0–23.9)
		Central Asia	—	—	17.3 (12.0–24.1)
U7b	T10084C	All together	10.0 (7.3–12.7)	6.7 (3.7–9.8)	10.1 (7.3–13.1)
		Near East	—	—	11.6 (8.2–15.5)
		South Asia	—	—	10.9 (7.1–15.5)
		Europe	—	—	9.6 (6.3–13.3)
U7c	C14131T	South Asia	5.9 (2.8 to 9.1)	7.2 (1.0 to 13.3)	—
U7d	T11365C, C16150T	Near East	7.9 (1.7 to 14.4)	7.9 (−2.9 to 18.7)	—
U7e	G1709A	Near East	2.1 (−1.0 to 5.2)	1.6 (−1.5 to 4.7)	—
U7f	G13368A, G16390A	Near East	9.7 (2.9 to 16.8)	5.3 (−2.5 to 13.0)	—

Mutations were scored relative to the root of haplogroup U. Coalescence times were estimated with three methods – ρ whole-mtDNA clock, ρ synonymous clock, and Bayesian estimation. They are expressed in thousand years ago. 95% confidence intervals for ρ-based estimates as well as 95% HPD intervals for BEAST estimates are given in parentheses. For U7, U7a, and U7b Bayesian analyses were carried out also with region-specific sequences.
