# Supplementary material for: Origin and spread of human mitochondrial DNA haplogroup U7
Source: Sci Rep. 2017 Apr 7;7:46044. doi: 10.1038/srep46044 (PMC5384202; doi:10.1038/srep46044)
Supplement: Supplementary Figure and Table Legends [file srep46044-s1.doc]

**Supplementary Figure and Table Legends**

**Origin and spread of human mitochondrial DNA haplogroup U7**

Hovhannes Sahakyan,^1,2,†^,* Baharak Hooshiar Kashani,^3,43,†^ Rakesh Tamang,^4^ Alena Kushniarevich,^1,5^ Amirtharaj Francis,^6^ Marta D Costa,^7,8^ Ajai Kumar Pathak,^1,9^ Zaruhi Khachatryan,^2^ Indu Sharma,^10^ Mannis van Oven,^11^ Jüri Parik,^1,9^ Hrant Hovhannisyan,^2,12^ Ene Metspalu,^1,9^ Erwan Pennarun,^1^ Monika Karmin,^1^ Erika Tamm,^1,9^ Kristiina Tambets,^1^ Ardeshir Bahmanimehr,^2,44^ Tuuli Reisberg,^1,9^ Maere Reidla,^1,9^ Alessandro Achilli,^3^ Anna Olivieri,^3^ Francesca Gandini,^3,45^ Ugo A. Perego,^3^ Nadia Al-Zahery,^3^ Massoud Houshmand,^13^ Mohammad Hossein Sanati,^13^ Pedro Soares,^8,14^ Ekta Rai,^10^ Jelena Šarac,^1,15^ Tena Šarić,^1,15^ Varun Sharma,^10^ Luisa Pereira,^8,16^ Veronica Fernandes,^7,8,16^ Viktor Černý,^17^ Shirin Farjadian,^18^ Deepankar Pratap Singh,^6^ Hülya Azakli,^19^ Duran Üstek,^20^ Natalia Ekomasova (Trofimova),^1,21,22^ Ildus Kutuev,^1,21^ Sergei Litvinov,^1,21^ Marina Bermisheva,^21^ Elza K Khusnutdinova,^21,22^ Niraj Rai,^6^ Manvendra Singh,^6^ Vijay Kumar Singh,^6^ Alla G. Reddy,^6^ Helle-Viivi Tolk,^1^ Svjetlana Cvjetan,^15,23,24^ Lovorka Barac Lauc,^15,25^ Pavao Rudan,^15,26^ Emmanuel N. Michalodimitrakis,^27^ Nicholas P. Anagnou,^28,29^ Kalliopi I. Pappa,^29,30^ Maria V. Golubenko,^31^ Vladimir Orekhov,^32^ Svetlana A Borinskaya,^32^ Katrin Kaldma,^1,46^ Monica A Schauer,^33^ Maya Simionescu,^33^ Vladislava Gusar,^34,47^ Elena Grechanina,^34^ Periyasamy Govindaraj,^6^ Mikhail Voevoda,^35,36,37^ Larissa Damba,^35^ Swarkar Sharma,^10^ Lalji Singh,^6,48^ Ornella Semino,^3^ Doron M Behar,^1,38^ Levon Yepiskoposyan,^2^ Martin B Richards,^7,39^ Mait Metspalu,^1^ Toomas Kivisild,^1,9,40^ Kumarasamy Thangaraj,^6^ Phillip Endicott,^41^ Gyaneshwer Chaubey,^1^ Antonio Torroni,^3^ and Richard Villems,^1,9,42^

^1^Evolutionary Biology Group, Estonian Biocentre, Tartu 51010, Estonia

^2^Laboratory of Ethnogenomics, Institute of Molecular Biology of National Academy of Sciences, Yerevan 0014, Armenia

^3^Dipartimento di Biologia e Biotecnologie "L. Spallanzani", Università di Pavia, Pavia 27100, Italy

^4^Department of Zoology, University of Calcutta, Kolkata 700 019, India

^5^Institute of Genetics and Cytology, National Academy of Sciences, Minsk 220072, Belarus

^6^CSIR-Centre for Cellular and Molecular Biology, Hyderabad 500 007, India

^7^Faculty of Biological Sciences, University of Leeds, Leeds LS2 9JT, UK

^8^Instituto de Patologia e Imunologia Molecular da Universidade do Porto (IPATIMUP), Porto 4200-135, Portugal

^9^Department of Evolutionary Biology, Institute of Molecular and Cell Biology, University of Tartu, Tartu 51010, Estonia

^10^Human Genetics Research Group, Department of Biotechnology, Shri Mata Vaishno Devi University, Katra 182320, India

^11^Utrecht 3523 GN, The Netherlands

^12^Russian-Armenian University, Yerevan 0051, Armenia

^13^Department of Medical Genetics, National Institute of Genetic Engineering and Biotechnology, Tehran 14965/161, Iran

^14^Departamento de Biologia, CBMA (Centro de Biologia Molecular e Ambiental), Universidade do Minho, Braga 4710-057, Portugal

^15^Institute for Anthropological Research, Zagreb 10000, Croatia

^16^Instituto de Investigação e Inovação em Saúde, Universidade do Porto (i3S), Porto 4200-135, Portugal

^17^Department of Anthropology and Human Genetics, Faculty of Science, Charles University, Prague 128-43, Czech Republic

^18^Department of Immunology, Allergy Research Center, Shiraz University of Medical Sciences, Shiraz 71348-45794, Iran

^19^Genetic Department, Institute of Experimental Medicine, Istanbul University, Istanbul 33326, Turkey

^20^Department of Medical Genetics and REMER, Faculty of Medicine, Medipol University, Istanbul, 34810 Turkey

^21^Institute of Biochemistry and Genetics, Ufa Scientific Center of the Russian Academy of Sciences, Ufa 450054, Russia

^22^Department of Genetics and Fundamental Medicine of Bashkir State University, Ufa 450076, Russia

^23^Department of Molecular Biology, Ruđer Bošković Institute, Zagreb 10000, Croatia

^24^Mediterranean Institute for Life Sciences, Split 21000, Croatia

^25^Croatian Science Foundation, Zagreb 10000, Croatia

^26^Anthropological Centre of the Croatian Academy of Sciences and Arts, 10000 Zagreb, Croatia

^27^Department of Forensic Sciences and Toxicology, University of Crete, School of Medicine, Heraklion 71110, Greece

^28^Laboratory of Biology, University of Athens, School of Medicine, Athens 115 27, Greece

^29^Foundation for Biomedical Research of the Academy of Athens (IIBEAA), Athens 115 27, Greece

^30^First Department of Obstetrics and Gynecology, University of Athens, School of Medicine, Athens 115 27, Greece

^31^Institute of Medical Genetics, Tomsk Research Center, Russian Academy of Medical Sciences, Tomsk 634050, Russia

^32^Vavilov Institute of General Genetics, Russian Academy of Sciences, Moscow 119333, Russia

^33^Institute of Cellular Biology and Pathology “Nicolae Simionescu”, Bucharest PO Box 35-14, Romania

^34^Kharkiv Specialized Medical Genetic Centre (KSMGC), Kharkiv 61022, Ukraine

^35^Institute of Internal and Preventive Medicine, SB RAS, Novosibirsk 630089, Russia

^36^Institute of Cytology and Genetics SB RAS, Novosibirsk 630090, Russia

^37^Novosibirsk State University, Novosibirsk 630090, Russia

^38^Clalit National Cancer Control and Personalized Medicine Program, Carmel Medical Center, Haifa 3436212, Israel

^39^Department of Biological Sciences, School of Applied Sciences, University of Huddersfield, Huddersfield HD1 3DH, United Kingdom

^40^Department of Archaeology and Anthropology, University of Cambridge, Cambridge CB2 1QH, United Kingdom

^41^Musée de l'Homme, Paris 75116, France

^42^Estonian Academy of Sciences, Tallinn 10130, Estonia

^43^Present address: Institute for Medical Immunology, Université Libre de Bruxelles, Gosselies B-6041, Belgium

^44^Present address: Thalassemia and Haemophilia Genetic PND Research Center, Dastgheib Hospital, Shiraz University of Medical Sciences, Shiraz 71456-83769, Iran

^45^Present address: Department of Biological Sciences, School of Applied Sciences, University of Huddersfield, Huddersfield HD1 3DH, United Kingdom

^46^Present address: Department of Zoology, Institute of Agricultural and Environmental Sciences, Estonian University of Life Sciences, Tartu 51014, Estonia

^47^Present address: Research Center for Obstetrics, Gynecology and Perinatology, Moscow 117997, Russia

^48^Present address: Genome Foundation, Hyderabad 500 076, India

^†^These authors contributed equally to this work

*Corresponding author: E-mail: [johannes@ebc.ee](mailto:johannes@ebc.ee)

**Supplementary Figure S1**

Maximum-Parsimony Phylogenetic Tree of U7 Mitogenomes.

Mutations were scored relative to the Reconstructed Sapiens Reference Sequence (RSRS)^1^. Nucleotide changes are explicitly indicated for every position. Uppercase indicates a transition, while lowercase indicates a transversion, and "d” indicates a deletion. Insertions are written with position number followed by .XN where X corresponds to number of inserted nucleotides and N corresponds to the actual nucleotide(s). Heteroplasmies are indicated by their respective IUPAC codes. Synonymous (s), non-synonymous (ns), non-coding (nc) mutations and heteroplasmies are in black, red, blue and purple color, respectively. Recurrent mutations are underlined. Back-mutations are depicted by a “!” mark after the resulting nucleotide, double back-mutations are depicted with a “!!” mark after the resulting nucleotide. For mitogenomes downloaded from the National Center for Biotechnology Information (NCBI) website, GenBank numbers are provided. For samples from the HGDP-CEPH Human Genome Diversity Cell Line Panel, HGDP numbers are provided. Shadowed sequences indicate: dark blue – Near East and North Africa, light blue – Europe, dark green – India, light green – Pakistan and Pashtuns from Afghanistan, orange – Central, East, Southeast Asia; and Siberia. The ρ time estimates are shown on the right of clade labels according to the following color code: ρ whole-mtDNA clock estimate in green; ρ synonymous clock estimate in black. 95% confidence intervals are provided in parentheses. N – North, NW – Northwest, NE – Northeast, NC – North-central, C – Central, S – South, SW – Southwest, SE – Southeast, SC – South-central, E – East.

**Supplementary Table S1**

Control-Region Mutational Motifs of U7 MtDNAs.

Mutations are scored relative to rCRS^2,3^. Transitions are denoted with position numbers only, and transversions are denoted by exact nucleotides after position numbers. Deletions are indicated by a "d" after the position numbers, while insertions are indicated with position numbers followed by .XN where X corresponds to the number of inserted nucleotides and N corresponds to the actual nucleotide(s). Heteroplasmies are reported according to IUPAC codes.

**Supplementary Table S2**

List of All U7 Complete Mitogenome Sequences Considered in This Study.

Mutations were scored relative to both rCRS^2,3^ and RSRS^1^. When using rCRS as a reference, transitions are denoted with position numbers only, and transversions are denoted by exact nucleotides after position numbers. When using RSRS as a reference, nucleotide changes are explicitly indicated, with the resulting nucleotide denoted by uppercase in case of transitions and by lowercase in case of transversions. In both cases, deletions are indicated by a "d” after the position numbers, while insertions are indicated with position numbers followed by .XN where X corresponds to the number of inserted nucleotides and N corresponds to the actual nucleotide(s). Heteroplasmies are reported according to IUPAC codes.

**Supplementary Table S3**

Population Frequencies of U7 and its Subclades.

*n1* – sample size, *n2* – number of U7 mtDNAs. Values in columns E-AJ are absolute numbers. Values in columns F, I, J, W, and Y (in red color) are the sums of those in the respective subclades.

**Supplementary Table S4**

Coalescence Time Estimates and Defining Mutations of Haplogroup U7 and its Subclades.

Mutations were scored relative to the root of haplogroup U. Coalescence times were estimated with three methods – ρ whole-mtDNA clock, ρ synonymous clock, and Bayesian estimation. They are expressed in thousand years ago. 95% confidence intervals for ρ-based estimates as well as 95% HPD intervals for BEAST estimates are given in parentheses. For U7, U7a, and U7b, Bayesian analyses were carried out also with region-specific sequences.

**References**

1. Behar, D. M. *et al.* A ‘Copernican’ reassessment of the human mitochondrial DNA tree from its root. *Am. J. Hum. Genet.* **90,** 675–684 (2012).

2. Anderson, S. *et al.* Sequence and organization of the human mitochondrial genome. *Nature* **290,** 457–465 (1981).

3. Andrews, R. M. *et al.* Reanalysis and revision of the Cambridge reference sequence for human mitochondrial DNA. *Nat. Genet.* **23,** 147–147 (1999).
